# Compartment Syndrome following Intramedullary Nail Fixation in Closed Tibial Shaft Fractures

**DOI:** 10.5704/MOJ.2307.005

**Published:** 2023-07

**Authors:** E Chng, M Satkunanantham, YC Kang, S Sechachalam

**Affiliations:** 1 Department of General Medicine, Khoo Teck Puat Hospital, Singapore; 2 Department of Hand and Reconstructive Microsurgery, Tan Tock Seng Hospital, Singapore; 3 Department of Hand and Reconstructive Microsurgery, Singapore General Hospital, Singapore

**Keywords:** compartment syndrome, tibial shaft fracture, intramedullary nail, reconstruction, tendon transfer

## Abstract

**Introduction:**

Compartment syndrome complicating intramedullary nailing of closed tibia fractures has been described as early as the 1980s, but currently remains less described in literature compared to compartment syndrome directly following trauma. This study aims to review this potentially disabling complication and highlight the importance of timely diagnosis and management of compartment syndrome following fracture fixation, not just after fracture itself, via a review of three cases.

**Material and methods:**

A retrospective study of a series of three cases was conducted. The type of fracture, wait time to fixation, surgery duration, reaming, size of nail implant used, tourniquet time, and surgical technique were recorded. Time to diagnosis of compartment syndrome, compartment pressure if available, extent of muscle necrosis, reconstructive procedures performed, and post-operative complications were analysed.

**Results:**

The three cases following high-energy trauma from road traffic accidents presented from January to May 2010. Compartment syndrome was diagnosed clinically for all cases, between one to six days post-operatively and supported by elevated compartment pressure measurements in two of the three cases.

**Conclusion:**

This study advocates thorough clinical monitoring and maintaining strong clinical suspicion of compartment syndrome in patients even after intramedullary nail fixation of tibial shaft fractures to achieve timely limb-salvaging intervention. While intercompartmental pressure can be used to aid in diagnosis, we do not advise using it in isolation to diagnose compartment syndrome. Tendon transfer improves functional mobility and provides a good result in patients with severe muscle damage, while skin grafting sufficient in patients with minimal muscle damage.

## Introduction

Compartment syndrome is a condition in which elevated tissue pressures within the compartment causes circulatory embarrassment and eventually tissue necrosis if not treated early^1^. It is a surgical emergency, commonly following high energy, traumatic accidents to long bones. Compartment syndrome is a clinical diagnosis, usually presenting with pain and palpable tense swollen compartment, worse on stretching of the compartment. Diagnosis may be supported by measurement of intracompartmental pressures where perfusion pressures <30mmHg or absolute pressure of >30mmHg. Following urgent fasciotomy and debridement, common soft tissue reconstruction and resurfacing options include tendon lengthening, tendon transfer, flaps, and skin grafts^2^.

While compartment syndrome after tibial fractures has been generally well studied, literature on compartment syndrome following surgical management, specifically intramedullary nailing, has been few and far between, with around 10 papers since 1980s, with a reported incidence ranging from 1.4% to 7%^3-13^. Comparatively, the reported rates of compartment syndrome following tibia fractures ranges from 2% to 9%^14-16^.

This study aims to review this potentially disabling complication and highlight the importance of timely diagnosis and management of compartment syndrome following fracture fixation, not just after fracture itself, via a review of three cases.

## Materials and Methods

A retrospective review of three cases of compartment syndrome that developed after intramedullary nailing of closed tibial fractures, was performed. The type of fracture, wait time to fixation, surgery duration, reaming, size of nail implant used, tourniquet time, and surgical technique were recorded. Time to diagnosis of compartment syndrome, compartment pressure if available, extent of muscle necrosis, reconstructive procedures performed, and post-operative complications were analysed. Post-operative follow-up with regards to wound healing, gait, ankle and toe range of motion were also documented. Radiograph images were obtained from the electronic medical records system. The study was approved by our domain specific review board.

## Results

**Fig 1:**
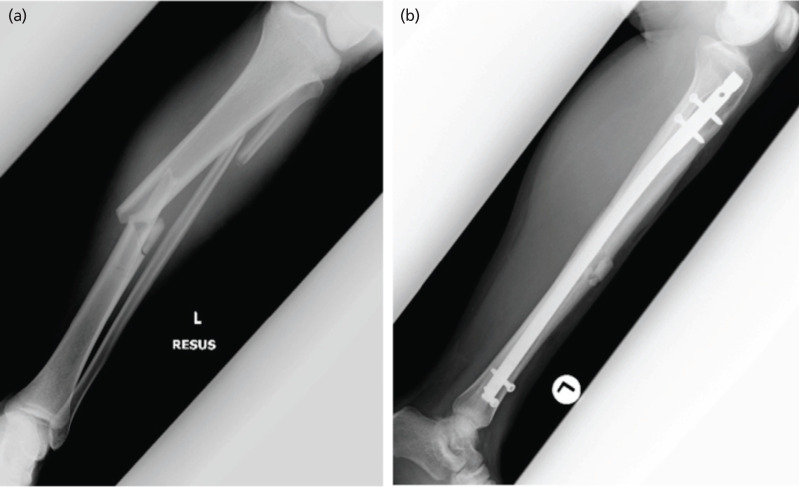
Radiograph images of Case 1. (a) Initial injury on arrival. (b) Post-operative follow-up.

**Fig 2:**
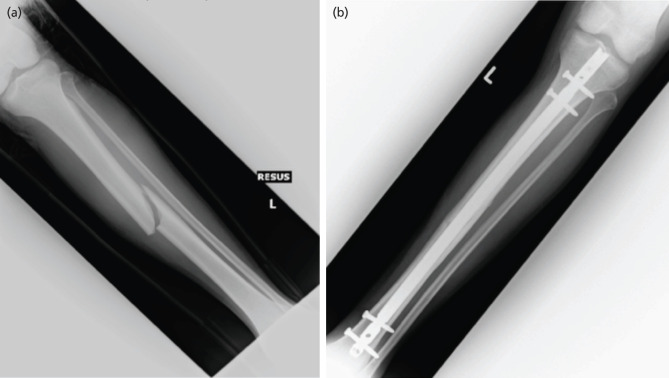
Radiograph images of Case 2. (a) Initial injury on arrival. (b) Post-operative follow-up.

**Fig 3:**
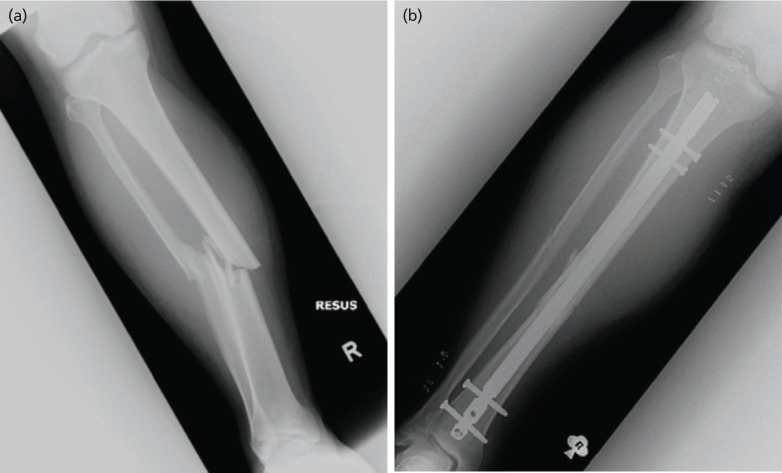
Radiograph images of Case 3. (a) Initial injury on arrival. (b) Post-operative follow-up.

The three cases presented from January to May 2010. All three cases were males, aged 21 to 44 years (mean 29 years), who had sustained closed tibia shaft fractures after high-energy trauma from road traffic accidents. Pertinent case details are presented in Table I and II, and radiograph images are shown (Fig. 1, 2 and 3).

**Table I:** Summary of presentation and fracture fixation of 3 cases in the retrospective study

Case No.	Age/ years	Presenting injury	Side of injury	Wait time to nailing / hours	Surgery duration/ minutes	Reaming size	Nail size	Tourniquet time/ minutes
1	21	Tibia and fibula shaft comminuted fracture	Left	24	165	13	12	120
2	23	Tibia midshaft fracture	Left	11	100	12.5	11	NA
3	44	Tibia midshaft and fibula comminuted fracture	Right	17	115	13	12	120

Abbreviation – NA: not applicable

**Table II:** Summary of compartment syndrome diagnosis, treatment, and recovery of 3 cases in the retrospective study

Case No.	Compartment syndrome Symptoms	Objective findings supporting compartment syndrome	Fasciotomy performed/ hours after fixation	Fasciotomy findings	Reconstruction	Post-operative complications	Outcome at 4 months	Others
1	Painless mild left calf swelling on POD3 Foot drop on POD4	Electromyography: Peroneal neuropathy	144	Ischaemic anterior compartment muscles with hematoma	Split TP to TA, EDL and EHL tendon transfer Split skin graft	NA	Active ankle dorsiflexion to neutral, plantarfl exion to 30°, toe extension	Sensation impaired over lateral and posterior calf
2	Pain over left calf Inability to actively dorsiflex foot	Compartment pressures: Anterior: 95mmHg Lateral: 40mmHg Posterior: 40mmHg	32	Ischaemic anterior and part of lateral compartment muscles	Split TP to TA, EDL and EHL tendon transfer Primary closure	Infection of EDL component EDL taken down, split skin graft performed	Active ankle dorsiflexion 10°, plantarfl exion to 30°	NA
3	Excessively swollen and tender calf post-operatively	Compartment pressures: Anterior: 32mmHg Lateral: 7mmHg Posterior: 12mmHg	24	Bruised posterior compartment muscles	Split skin graft	NA	Active ankle dorsiflexion 30°, plantarflexion to 40°	NA

Abbreviations - POD: post-operative day, TP: tibialis posterior, TA: tibialis anterior, EDL: extensor digitorum longus, EHL: extensor hallucis longus, NA: not applicable

Prior to surgery, fractures were stabilised temporarily in a backslab. Surgical fixation of the tibia fractures using reamed locking intramedullary tibial nails took place within 24 hours of the respective injuries. The average duration of the surgical fixation was 126±34 minutes, and Cases 1 and 3 each had a 120-minute tourniquet time recorded. No tourniquet was used for Case 2. The tourniquet was temporarily released during reaming for Case 1 for 53 mins. Despite pre-operative swelling documented in Case 3, preoperative compartment pressures were between 28-30mmHg for each of the four leg compartments, hence prophylactic fasciotomy during surgical fixation was not performed.

Compartment syndrome was diagnosed clinically for all cases, with the symptoms and objective findings summarised in Table I. Diagnosis was made between one to six days postoperatively and supported by elevated compartment pressure measurements in two of the three cases.

In each of the three cases, fasciotomy was done at an average of 56 hours (range 5-144h) post fixation. Subsequently, tendon transfer was performed for Case 1 and Case 2, while Case 3 required skin grafting only. Additional debridement and skin grafting was required for Case 2 after infection of Extensor Digitorum Longus (EDL) component, two weeks after primary closure of the tendon transfer wound.

## Discussion

The diagnosis of compartment syndrome is notoriously difficult^17^. A combination of clinical signs and investigations have been described to assist in the diagnosis of compartment syndrome.

In our study, there was a wide time range of 5 to 144 hours from time from post fixation to fasciotomy. Case 1 had the most delay in diagnosis of compartment syndrome compared to the other two cases, leading to a longer-to-fasciotomy time, and more extensive reconstruction required. We attribute this delay to an atypical presentation of compartment syndrome where the patient complained of minimal pain symptoms, with minimal swelling on postoperative day 4, then foot drop on post-op day 4, worked up with EMG to show peroneal neuropathy and reached the diagnosis. In addition, we noted that two of our three cases had use of tourniquet during the primary surgery. Tourniquets were used in accordance with literature to control bleeding to improve the surgical field of view in fracture fixation surgeries and aid in reducing operative time^18-19^. Tourniquet use has not been established as statistically significant risk factor for post fixation compartment syndrome thus far. Future research should explore this issue, so that if it is established it as a risk factor, surgeons can take this into consideration when deciding on use of tourniquet, with careful weighing of risk and benefits, and also increase vigilance post-operatively, in cases where tourniquets are used.

Pain with passive stretch of the affected compartment is the key early clinical sign in the diagnosis of compartment syndrome, with the classical findings of pallor, paraesthesia, paralysis and pulselessness developing later, as seen in Cases 2 and 3s. The diagnosis of compartment syndrome may also be augmented with the use of the compartment pressure monitor, as was done in Cases 2 and 3. An intracompartmental pressure of 30mmHg or differential pressure (the difference between diastolic pressure and intracompartmental pressure) of 30mmHg, has been recommended to be the threshold for fasciotomy in the lower limb^20^. Young age (less than 35 years) and male gender have been shown to be risk factors for post traumatic acute compartment syndrome, and such patients who are at risk for developing this complication warrant increased vigilance and suspicion for compartment syndrome^17^. On the contrary, mechanism of injury, type of trauma, anatomical site of fracture on tibia, type of fracture (open vs closed) and fixation methods have not been found to be statistically significant risk factors.

Clinical diagnosis of compartment syndrome postoperatively is even more complex because swelling is normal in the post-operative recovery phase. The administration of post-operative analgesia such as patient-controlled analgesia also confounds the symptoms of pain^19,21^. This could attribute to the longer time to diagnosis for Case 1. At the same time, as raised compartment pressures are frequently seen in patients with tibial shaft fractures, compartment pressure is unable to be used in isolation to diagnose compartment syndrome^22^.

Compartment syndrome may develop post-operatively due to the rise of compartment pressures during traction and reaming, early surgery, delayed surgery and the use of tourniquet^19^. No correlation has been found between the type of fracture, degree of trauma, presence of a new common peroneal nerve lesion, fracture treatment delay, or prior closed reduction attempts, and the development of compartment syndrome after tibia nailing^10,23^. More robust studies may unearth possible predisposing risk factors for post fixation compartment syndrome. Nevertheless, patients should receive decompressive fasciotomy as soon as possible to prevent further injury to muscles and nerves, failing which, poorer outcomes would be likely to ensue^11^, as seen from Case 1 where there is permanent sensation deficit despite fasciotomy done on post-operative day 6.

**Fig 4:**
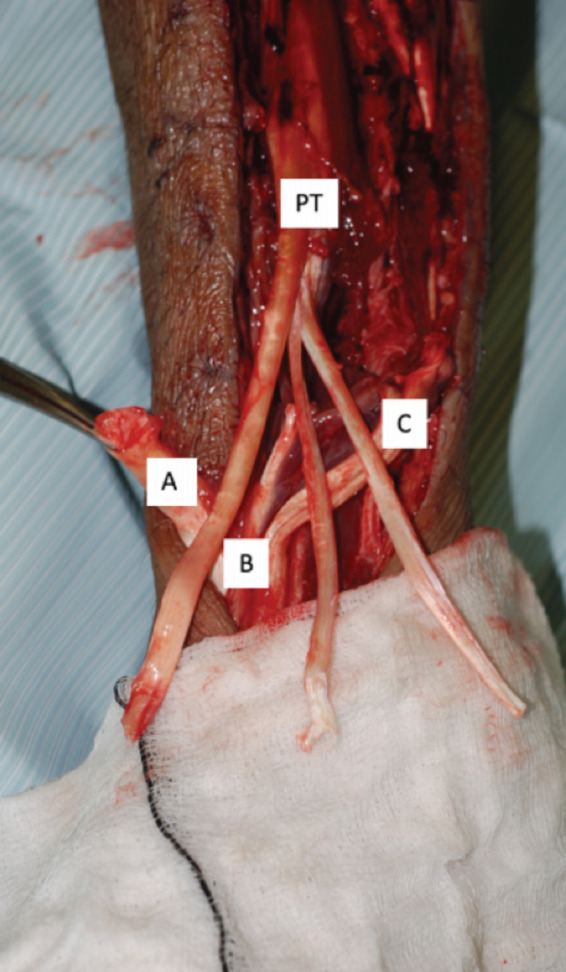
Intra-operative pictures taken during tendon transfer surgery for Case 2. (A): Tibialis Anterior tendon. (B): Extensor Hallucis Longus tendon. (C): Extensor Digitorum Longus tendon. (PT): Tibialis Posterior tendon split into three distally.

The need for the reconstruction arises after fasciotomy, when extensive musculature loss causes functional deficits which impact the mobility and quality of life of patients^24,25^. Flexible deformities can be treated with tendon lengthening or tendon transfer (Fig. 4), while progressive contractures may be subject to tenotomy, capsular release and excision of scarred tissue, as in Cases 1 and 2^26,27^.

## Conclusion

Our study advocates thorough clinical monitoring for pain, swelling, and neurovascular compromise, and maintaining strong clinical suspicion of compartment syndrome in patients even after intramedullary nail fixation of tibial shaft fractures to achieve timely limb-salvaging intervention. While intercompartmental pressure can be used to aid in diagnosis, we do not advise using it in isolation to diagnose compartment syndrome. Our review shows that patients need not be left with permanent sequelae as appropriate management such as early fasciotomy or tendon transfer could decrease functional deficits.
